# Case Report: A COVID-19 Patient Presenting with Mild Rhabdomyolysis

**DOI:** 10.4269/ajtmh.20-0583

**Published:** 2020-06-19

**Authors:** Betul Borku Uysal, Hande Ikitimur, Serap Yavuzer, Mehmet Sami Islamoglu, Mahir Cengiz

**Affiliations:** 1 Department of Internal Medicine, Medical Faculty, Biruni University, Istanbul, Turkey;; 2 Department of Pulmonary Diseases, Medical Faculty, Biruni University, Istanbul, Turkey

## Abstract

The news was reported from the Wuhan region of China about a novel corona virus in the end of 2019. After spreading around the world, a pandemic was declared by the WHO. Depending on the different involvement of the disease, the most common symptoms are fever, cough, and dyspnea. However, some indeterminate symptoms that make diagnosis difficult, such as myalgia and fatigue, can also be seen alone, without the typical clinical picture. We describe a patient with COVID-19 pneumonia, the only complaint of which is myalgia, and the first diagnosis is mild rhabdomyolysis. The patient had no evidence or history other than viral infection that could explain muscle pain and also increased level of muscle enzymes. When mild rhabdomyolysis lack of myoglobinuria and complications was diagnosed, treatment-related rhabdomyolysis was also avoided as no treatment related to COVID-19 was initiated yet. Apart from the typical symptoms leading to the typical diagnosis of COVID-19 at the first admission, SARS-CoV-2 related with rhabdomyolysis should also be kept in mind.

## INTRODUCTION

Rhabdomyolysis is the breakdown of skeletal muscle by releasing its cellular content into the systemic circulation. The most common acquired causes are substance abuse (34%), medication (11%), trauma (9%), and epileptic seizures (7%).^1^ Less common causes include metabolic disorders, infections, local muscle ischemia, general muscle ischemia, prolonged immobility, exercise, and excessive heat.^1,2^ Classical clinical features are acute-onset myalgia, transient muscle weakness, and pigmenturia.^1,3^ Proposed diagnostic algorithm includes taking a detailed history asking for symptoms, to confirm the diagnosis of rhabdomyolysis by neurological examination, creatine phosphokinase (CK) and/or myoglobinuria measurement. The third step is to reveal the underlying cause of rhabdomyolysis.

The disease was designated as COVID-19 by the WHO in February 2020, and the pathogen caused by SARS-CoV-2 has spread out in the world.^4^ SARS-CoV-2 infection represents a spectrum of clinical severity. The most prevalent clinical manifestations are fever (88.7%, 95% CI: 84.5–92.9%), cough (57.6%, 95% CI: 40.8–74.4%), and dyspnea (45.6%, 95% CI: 10.9–80.4%).^5^ COVID-19 mostly affects the respiratory system, ranging from mild flu-like symptoms to severe pneumonia, but extra-respiratory multisystemic involvement has also been reported.^6^

Here, we describe a case report of a male patient who was first presented with rhabdomyolysis and was subsequently diagnosed with COVID-19.

## CASE PRESENTATION

A 60-year-old male patient was admitted with myalgia and fatigue for 2 days. The patient had no fever, cough, sore throat, chest tightness, and shortness of breath.

The patient had no previous history of chronic diseases such as kidney disease, hypertension, and an endocrine disorder; muscle diseases such as muscular dystrophy and neuromuscular disorders; or a statin class of drug use that could lead to muscle breakdown. There was no trauma and no history of medication. The patient reported no alcohol intake and smoking.

### Physical examination on admission.

Blood pressure was 120/70 mmHg, respiratory rate 18/minutes, heart rate 93/minutes, temperature 36.7°C, and arterial oxygen saturation 98% in room air. The patient was conscious and had a clear mind. He was in a regular heart rhythm without any obvious murmur. There were obvious moist crackles during auscultation of inferior lobes in the lung.

### Laboratory examinations.

Routine blood test results were as follows: white blood cells, 2.91 × 10^9^/L; neutrophils, 55.7%; lymphocytes, 30.2%; total number of lymphocytes, 0.88 × 10^9^/L; C-reactive protein (CRP), 35.1 mg/L; potassium, 4.3 mmol/L; sodium, 138 mmol/L; urea, 21 mg/dL; D-dimer, 210 ng/mL; alanine aminotransferase (ALT), 52 U/L; aspartate aminotransferase (AST), 117 U/L; lactate dehydrogenase (LDH), 575 U/L; ferritin, 428 ng/mL; international normalized ratio, 1.07; prothrombin time, 11 minutes; total bilirubin, 0.5 mg/dL; and direct bilirubin, 0.2 mg/dL. Cardiac acute damage marker values such as creatine kinase myocardial band (CK-MB: 3.80 U/L) and troponin (13.6 pg/mL) were among normal reference values at the time of hospital admission.

The patient’s baseline serum creatinine (Cr) level was 0.91 mg/dL and baseline serum CK level was 4,267 U/L. Changes in CK, CRP, AST, ALT, LDH, ferritin, and D-dimer during the patient’s hospitalization are presented in Table 1.

**Table 1:** Changes in biochemical markers during the patient’s hospitalization

April	Normal ranges	Creatine phosphokinase (U/L)	C-reactive protein (mg//L)	Aspartate aminotransferase (U/L)	Alanine aminotransferase (U/L)	Lactate dehydrogenase (U/L)	Ferritin (ng/mL)	D-dimer (ng/mL)
		30–200	0–5	5–38	5–45	125–220	21–274	0–500
10th		**4,267**	35	117	52	575	428	210
11th		**3,151**	62	124	65	691	599	350
12th		**2,307**	108	126	84	707	874	623
13th		**1,612**	127	137	102	781	1,688	905
14th		**776**	138	127	129	743	> 2,000	508
15th		**389**	70	94	134	639	> 2,000	380
16th		**191**	30	89	152	532	1,739	363
17th		**161**	22	95	208	526	1,348	342
18th		**101**	15	46	162	373	970	330
19th		**96**	14	29	113	337	608	312

Bold values indicate change of creatinine kinase, an important indicator of rhabdomyolysis.

Blood tests for rapid detection of influenza A, influenza B, respiratory syncytial virus, adenovirus, hepatitis A, and hepatitis B were all negative. Electrocardiogram was normal; no significant cardiac arrhythmia was detected.

### Clinical diagnosis.

**Figure 1.**
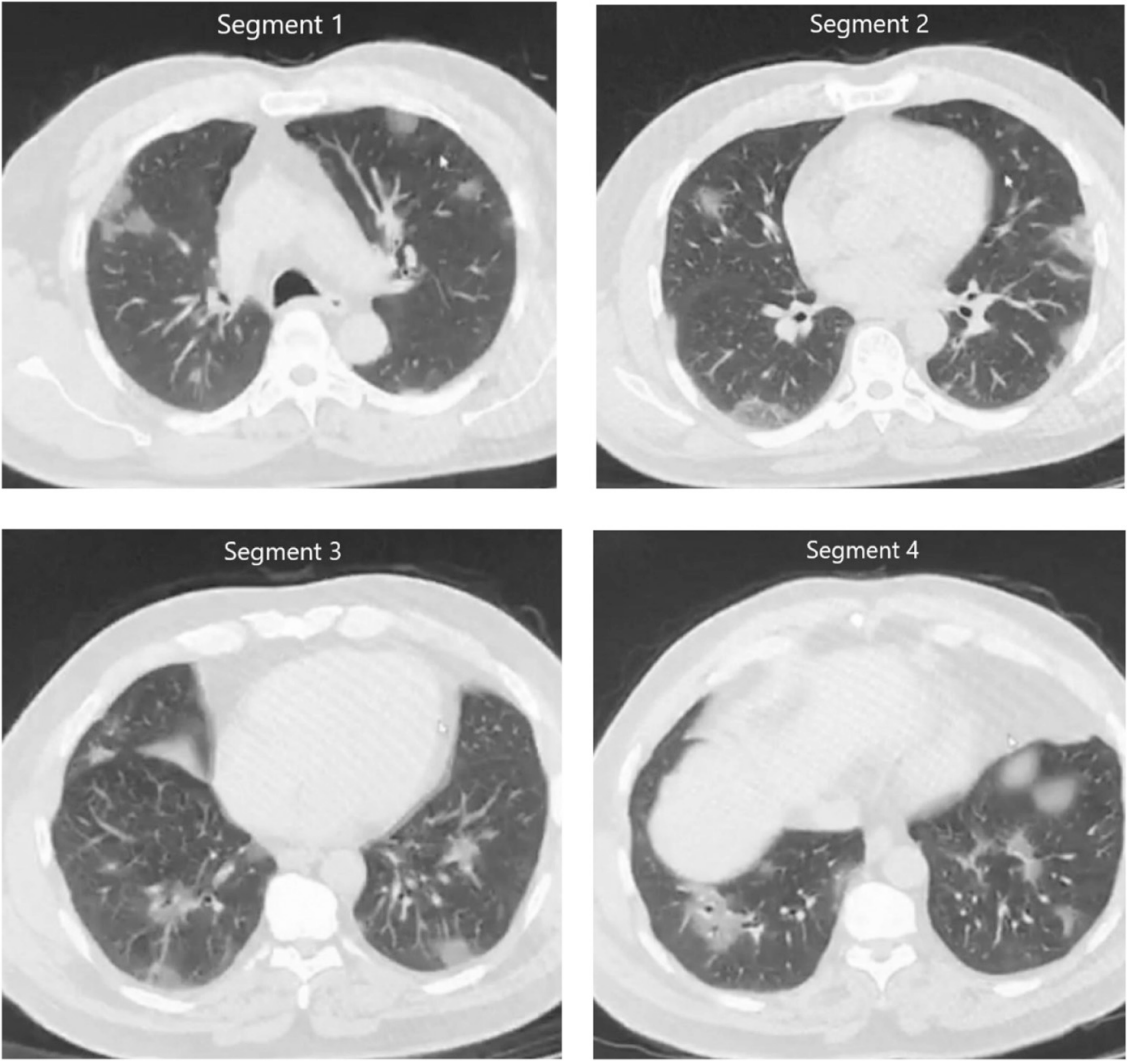
Ground-glass nodules are randomly distributed in the upper and lower lobes of the lungs and the pleural membrane in computed chest tomography.

The patient, who was admitted with frequent complaints of myalgia and fatigue, was diagnosed in the emergency department. Considering the clinical features, physical examination, and chest radiography, the patient was hospitalized with the diagnosis of suspected viral infection. However, considering COVID-19 infection to the epidemic area, atypical pneumonia virus infection had to be ruled out. On the same day, a chest computerized tomography (CT) scan was performed, and small ground-glass nodules were seen, suggesting viral pneumonia scattered across the two lungs (Figure 1). Two days later, a real-time reverse transcription–PCR (RT-PCR) analysis of the patient’s throat swab sample indicated SARS-CoV-2 infection.

### Treatment and follow-up.

We hospitalized the male patient diagnosed with COVID-19. When we evaluated according to the WHO classification, we found that our patient had severe pneumonia. During hospitalization, hydroxychloroquine (first day 2 × 400 mg loading + 4 days 2 × 200 mg maintenance), oseltamivir (2 × 75 mg, 5 days), and azithromycin (first day 1 × 500 mg loading + 4 days 1 × 250 mg) therapy were used as recommended in the COVID-19 guidelines.^7^ The patient was quickly treated with a combination of IV fluids and sodium bicarbonate for alkalization. After five days of treatment, the patient’s clinical condition deteriorated, and the patient’s respiratory rate was 30 breaths/min; severe respiratory distress developed, and SpO_2_ was ≤ 93% on room air. Favipiravir (first day 2 × 1,600 mg loading + 4 days 2 × 600 mg) antiviral treatment was added to the treatment of the patient, who was thought to develop severe pneumonia. The length of hospital stay of our patient was 10 days between April 9, 2020 and April 19, 2020. After 11 days of treatment, the patient’s symptoms improved significantly, and he recovered with a negative RT-PCR test.

## DISCUSSION

In rare COVID-19 cases, patients have no typical symptoms and can only be confirmed by RT-PCR testing and/or a CT scan. Therefore, clinicians should be aware of the atypical characteristics of SARS-CoV-2 infection, especially in patients with an epidemic contact history.^8^

When our case was admitted to the emergency room, he did not have any symptoms and contact history of SARS-CoV-2 infection. The only complaint of the patient was severe muscle aches. After the examinations in terms of muscle pain etiology, rhabdomyolysis was diagnosed. There was also no history of the comorbid disease and drug use that could cause rhabdomyolysis in the anamnesis of the patient. However, because of the epidemic, the diagnosis of COVID-19 was confirmed by RT-PCR after viral pneumonia was detected in the patient’s simultaneous radiographic examination. For all these reasons, we think that this COVID-19 case with rhabdomyolysis onset is unusual.

The risk of developing rhabdomyolysis after drug use is high.^3^ The development of rhabdomyolysis after the use of hydroxychloroquine^3^ and oseltamivir,^9^ which is also used in the treatment of COVID-19, has been demonstrated before the COVID-19 outbreak. In the literature, a case with COVID-19 diagnosis and rhabdomyolysis under treatment is presented.^10^ Our patient’s clinical presentation alone, without any treatment, was muscle pain and elevation of the enzyme due to muscle destruction, proved that rhabdomyolysis developed because of SARS-COV-2 infection. Although CK and liver enzymes were high after diagnosis of our case, hydroxychloroquine was added to the treatment from the first day and favipiravir as an antiviral treatment after the fifth day. Despite the worsening of the patient’s clinical condition on the fifth day of treatment, a marked decrease in CK was started to be observed. In accordance with the clinical course of rhabdomyolysis, the first muscle stimulation and CK decreased within 5–6 days after reaching the peak value independent of the patient’s clinical condition.

The major divergence in the diagnostic criteria is related to the time of muscle injury onset and the strength of clinical parameters. The degree of CK elevation is proportional to muscle injury. Approximately 2–12 hours after the onset of muscle injury, CK increases. A peak concentration occurs at 24–72 hours, and then CK declines to baseline values in the course of 3–5 days.^11^ In our case, no increase in Cr value and myoglobinuria developed.

We thought that the increase in liver enzymes two days after the application may be due to rhabdomyolysis or the hepatotoxicity of the drugs used in the treatment of COVID-19. Hepatic dysfunction occurs in 25% of patients with rhabdomyolysis. An important factor of pathogenesis is hepatic inflammation, which is triggered by proteases released from injured muscle.^12^

The most common cause of virus-associated rhabdomyolysis is influenza. In an adult study of 505 patients, high CK levels at admission were found to be associated with renal failure, dialysis requirement, mechanical ventilation, and prolonged hospital stay.^13^ There are 42 cases of virus-induced rhabdomyolysis documented in the literature; influenza accounted for 14 of 42 (33%) of these reported cases.^9^ In our case, viral infections, especially influenza, were investigated at the initial stage of diagnosis, and, then, COVID-19 was detected by the RT-PCR test.

In conclusion, fatigue and myalgia due to COVID-19 are quite common symptoms, and rhabdomyolysis is an important complication to be remembered in patients with severe symptoms. It should be kept in mind that the drugs we use for COVID-19 treatment can cause myopathy as a side effect. However, myositis secondary to infection, like as our case, reveals that treating the infection may improve the patient’s clinical and laboratory results, and not worsen the myositis picture.
